# Multi-scale ancient DNA analyses confirm the western origin of Michelsberg farmers and document probable practices of human sacrifice

**DOI:** 10.1371/journal.pone.0179742

**Published:** 2017-07-05

**Authors:** Alice Beau, Maïté Rivollat, Hélène Réveillas, Marie-Hélène Pemonge, Fanny Mendisco, Yohann Thomas, Philippe Lefranc, Marie-France Deguilloux

**Affiliations:** 1 De la Préhistoire à l’Actuel, Culture, Environnement, Anthropologie—UMR 5199, CNRS, Université de Bordeaux, Allée Geoffroy Saint-Hilaire, CS, Pessac cedex, France; 2 Centre d’Archéologie Préventive de Bordeaux Métropole, Direction des Bâtiments et Moyens, Esplanade Charles-de-Gaulle, Bordeaux cedex, France; 3 Institut National de Recherche en Archéologie Préventive, Centre Archéologique de Strasbourg, 10 rue d’Altkirch, Strasbourg, France; 4 Archéologie et Histoire Ancienne: Méditerranée/Europe–UMR 7044, Université de Strasbourg, Maison Interuniversitaire des Sciences de l’Homme d’Alsace, 5 Allée du Général Rouvillois, CS, Strasbourg cedex, France; University College Dublin, IRELAND

## Abstract

In Europe, the Middle Neolithic is characterized by an important diversification of cultures. In northeastern France, the appearance of the Michelsberg culture has been correlated with major cultural changes and interpreted as the result of the settlement of new groups originating from the Paris Basin. This cultural transition has been accompanied by the expansion of particular funerary practices involving inhumations within circular pits and individuals in “non-conventional” positions (deposited in the pits without any particular treatment). If the status of such individuals has been highly debated, the sacrifice hypothesis has been retained for the site of Gougenheim (Alsace). At the regional level, the analysis of the Gougenheim mitochondrial gene pool (SNPs and HVR-I sequence analyses) permitted us to highlight a major genetic break associated with the emergence of the Michelsberg in the region. This genetic discontinuity appeared to be linked to new affinities with farmers from the Paris Basin, correlated to a noticeable hunter-gatherer legacy. All of the evidence gathered supports (i) the occidental origin of the Michelsberg groups and (ii) the potential implication of this migration in the progression of the hunter-gatherer legacy from the Paris Basin to Alsace / Western Germany at the beginning of the Late Neolithic. At the local level, we noted some differences in the maternal gene pool of individuals in "conventional" *vs*. "non-conventional" positions. The relative genetic isolation of these sub-groups nicely echoes both their social distinction and the hypothesis of sacrifices retained for the site. Our investigation demonstrates that a multi-scale aDNA study of ancient communities offers a unique opportunity to disentangle the complex relationships between cultural and biological evolution.

## Introduction

The farming lifestyle, including innovative features such as pottery, polished stones and sedentism, appeared in the Near East from approximately 10,000 cal. BC. This process, called ‘Neolithization’ or Neolithic transition, is a fundamental period in human history since it has marked the transformation of the previous hunting-gathering system to a farming system and has shaped and revolutionized human societies as we know them today. The Neolithic diffusion towards Western Europe occurred through two major expansion waves: the continental wave following the Danube in Central Europe and the Mediterranean wave progressing along Mediterranean coastlines. The continental wave was associated with the Linearbandkeramik (LBK) culture and diffused from approximately 5,500 cal. BC in the middle of the Danube (Bohemia, Moravia, Hungary) and reached the Rhine around 5,300 cal. BC [1, 2].

During the last decade, ancient DNA (aDNA) data have provided new elements to delineate the processes involved in the Neolithic diffusion and population dynamics thereafter. Studies showed that farmers themselves, rather than just their culture, expanded from the Near East around 8,000 cal. BC, by detecting a very strong genetic differentiation between hunter-gatherers (H-G) and Europe’s first farmers [3–7]. Paleogenetic studies also confirmed that both different Neolithization waves ultimately derived from common roots in Anatolia [8, 9]. This spread from a common source is illustrated by the marked genetic homogeneity of early farmers, followed by a subtle genetic and temporal gradient across West Anatolia, the Carpathian Basin, Central Europe, and the Iberian Peninsula [8, 10–14]. The subsequent 2,000 years provided evidence for relatively little genetic change, except for a mild increase of H-G ancestry in farmers, attesting to a slow but steady introgression of forager lineages into farming communities during the Late Neolithic period, in regions documented such as Central Europe and the Iberian Peninsula [5, 11]. Nevertheless, signals of earlier admixture were detected farther west in the French territory, showing that processes of interactions between groups should have been highly variable at the regional scale [15].

In the Alsace region (north-western France), after a gap of two centuries (5,000–4,790 cal. BC), numerous cultures inherited from the LBK succeeded during the Middle Neolithic, including the Hinkelstein culture (4,790–4,730 cal. BC), Grossgartach culture (4,730–4,620 cal. BC), Roessen culture (4,620–4,460 cal. BC) and then the Bischheim and Bruebach-Oberbergen cultures at the end of the fifth millennium BC (4,460–4,240 cal. BC) [16]. The first mitochondrial DNA data (mtDNA) obtained from the Middle Neolithic Grossgartach / Roessen groups confirmed their cultural and genetic continuity with LBK groups from Central Europe [17]. Around 4,400 cal. BC, the Michelsberg culture appeared, named after the archaeological site of Michelsberg in the German state of Baden-Wurtemberg [18] and developing from central Germany to the Paris Basin (north of France). Simultaneously, the Munzingen culture appeared in South Alsace, mainly described through its ceramic remains. Several hypotheses concerning the origin of the Michelsberg culture have been offered, but the one prevailing today (and first introduced in 1959 [19]) considers an origin in the Paris Basin [20–22]. This "occidental hypothesis" was reinforced by the demonstration of an important rupture in pottery traditions between the previous Alsatian cultures and the Michelsberg, combined with the continuity of specific ceramic features with southwestern traditions (in the Paris Basin). The importance and the speed of the cultural changes perceived led the author to consider that major migration should have been implied in these processes [20, 22]. At present, no genetic evidence is available to support this "western hypothesis"; only one Michelsberg group has been genetically analyzed (Bruchsal-Aue in western Germany [23]; N = 9), and its small gene pool fitted with the Central European genetic picture [5, 11].

**Fig 1:**
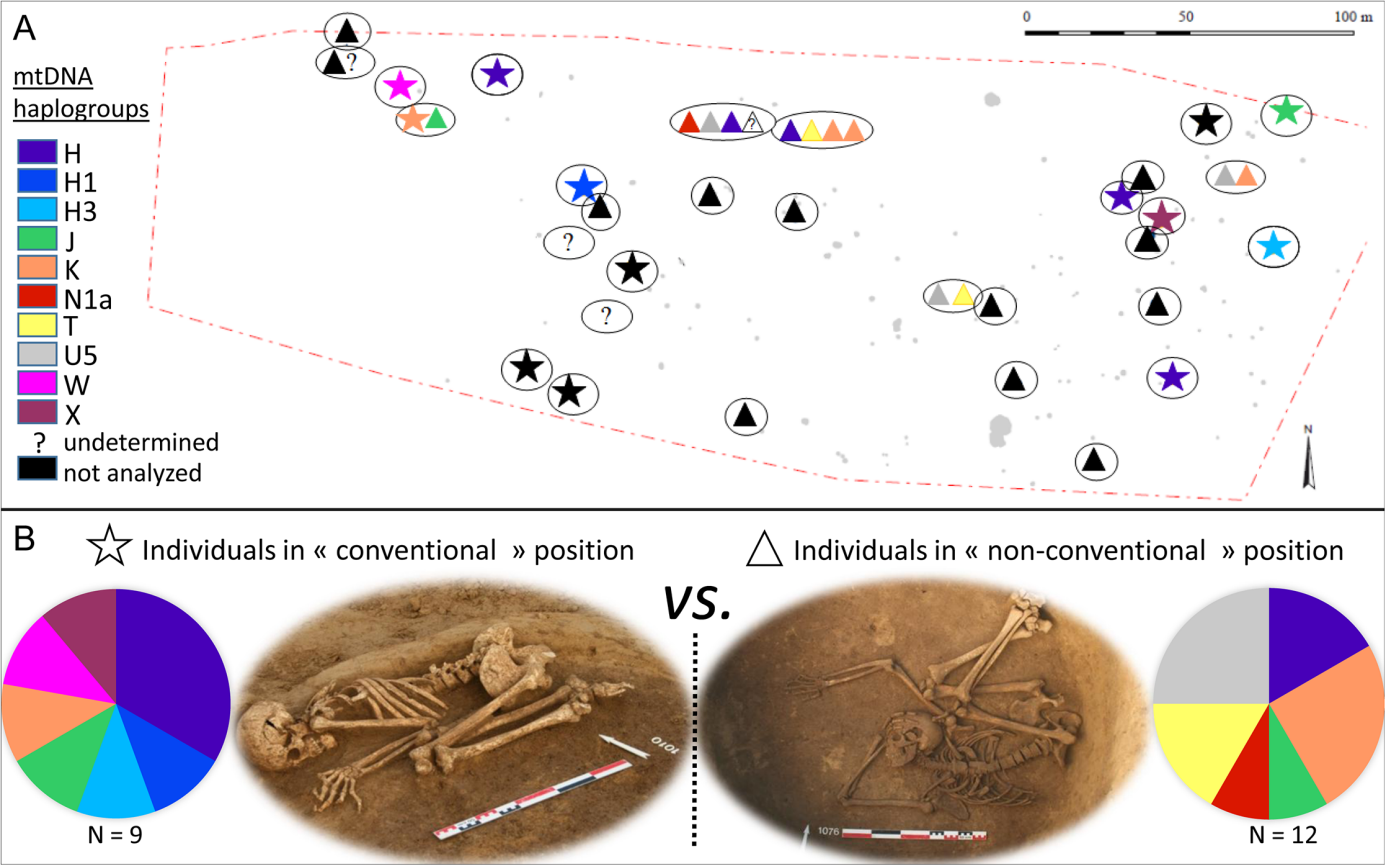
Spatial distribution of the mitochondrial (mtDNA) haplogroups within the Gougenheim site. (A) Inhumation repartition and mtDNA haplogroup distribution. Ellipses represent a circular pit; stars represent individuals in the "conventional position", whereas triangles represent individuals in the "non-conventional" position. (B) MtDNA haplogroup composition of "conventional" vs. "non-conventional" groups.

Interestingly, ceramic changes observed during the Michelsberg culture development have been accompanied by the apparition of particular funerary practices involving inhumations within circular pits. The mortuary practice of burying people in circular pits appeared simultaneously in the *Chasséen* culture in the south of France and in the epi-Lengyel Münchshöfen culture of Lower Bavaria [24]. In the current state of research, its diffusion through some Michelsberg groups originated from the Danube area—considering that Michelsberg and Münchshöfen are in close contact in central Lower Bavaria—and promptly extended toward the Rhine and until the middle Rhône valley, where it is strikingly illustrated in some late Chasséen or “la Roberte” contexts [25, 26]. To the east, the practice reaches the late Lengyel (Ludanice, Balaton-Lasinja), Baalberge and the Funnel Beaker culture. We find it again later, in the same area, in the Salzmünde and Boleraz/Baden cultures. The status of these peculiar pits, as well as the status of the individuals deposited in such structures, has been the subject of intense debate. Concerning the status of the pits, whereas C. Nickel proposed to not consider them as funerary pits (but more as marginalized practices [27]), J. Lichardus and J. Schweitzer viewed them as true graves within pits especially dug for this purpose or that might have had another function in the past (storage, habitation) [28, 29]. Concerning the status of the recovered individuals, the debate focused on the observation of two distinct inhumation positions described within the circulars pits: the “conventional” position (*CV*), where the subject is placed on its side, both superior and inferior members folded up against the chest, *vs*. the “non-conventional” position (*NCV*) where the bodies seem to have been thrown into the pits without any particular treatment (Fig 1B). Moreover, different categories of deposits could be depicted: (i) individual or multiple “conventional” deposits, (ii) individual or multiple “non-conventional” deposits and (iii) multiple “asymmetrical” deposits where both *CV* and *NCV* positions were found within the same structure. Such striking differences in funerary treatment led to the obvious question of the specific status of the individuals found in the *NCV* position. Thanks to the multiplication of the discoveries and with the assistance of ethnological examples, distinct interpretations have been proposed including (i) graves of "relegation", *i*.*e*., graves that are excluded from the funerary space [30], (ii) graves of "deprivation", where bodies are treated like waste [31], or (iii) practices involving some ritual killing divided between “funerary accompaniment” and sacrifice *stricto-sensu*. According to the last proposal, individuals found in the *NCV* position can be related to "accompaniment dead" as defined by A. Testart, *i*.*e*., individuals intentionally put to death upon the decease of a figure of the community (with special social significance) [32]. In this way, accompaniment dead could be spouses following their husbands in tombs, soldiers giving up the fight and accompanying their leader in death, subjects of a theocratic kingdom killed upon the decease of their king or, finally and most commonly in lineage societies, slaves accompanying their master in death. According to the sacrifice hypothesis, *i*.*e*., the ritual murder of individuals outside of any funerary framework, the human victim is the subject and the vehicle of a supernatural transaction that establishes a direct link between men and some deities or spirits (as regularly encountered in different past civilizations such as the Aztecs, Mayas, Ibos, and Dayaks) [24].

**Fig 2:**
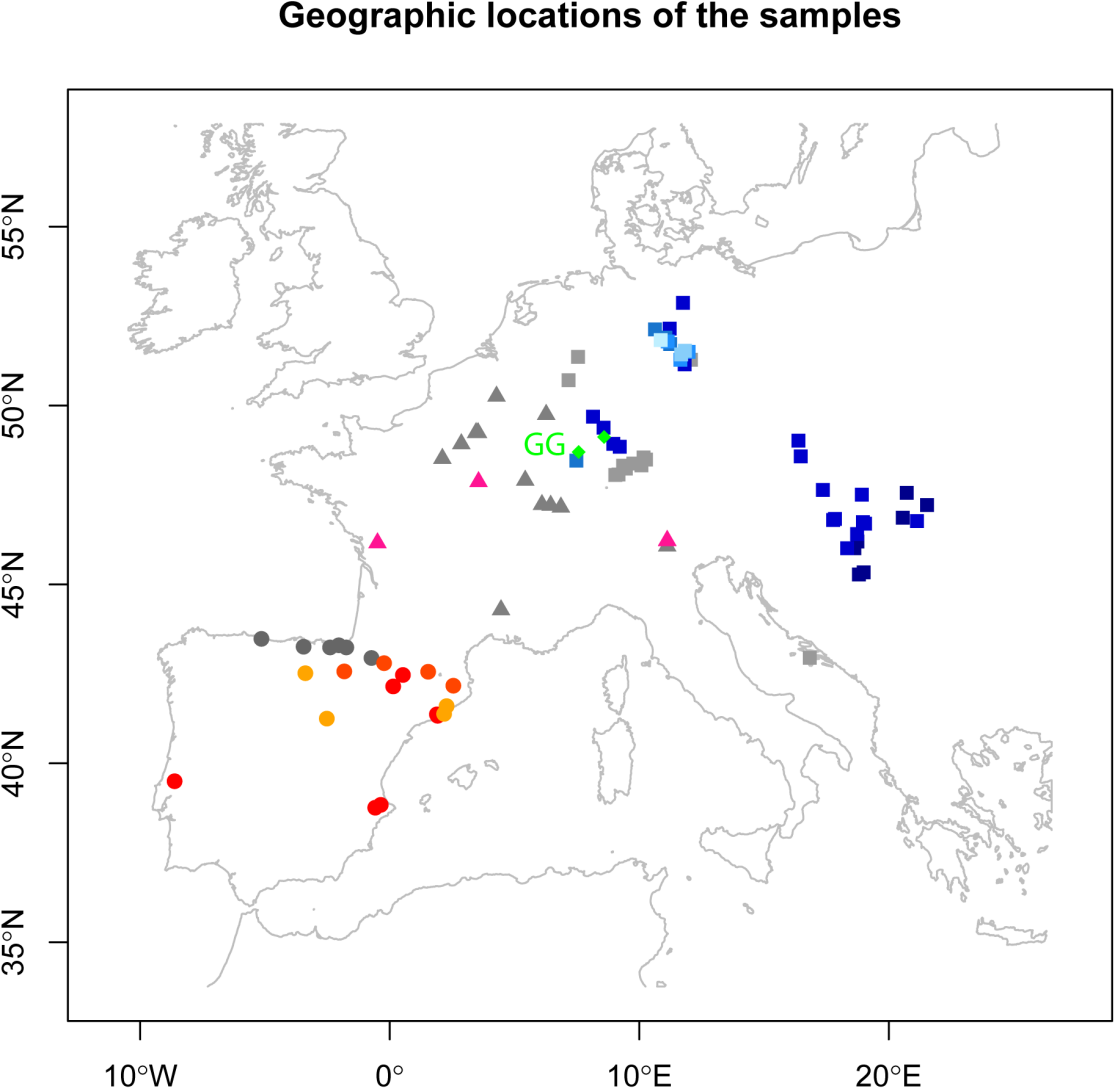
Localization of Gougenheim and of the sites anterior to 2,600 cal. BC. considered in the study. Central European groups (□); South European groups (○); French groups (Δ); MICH groups (◊). Hunter-gatherers (gray); Central European farmers (blue); South European farmers (red/orange); MNF farmers (pink); MICH farmers (green). Color gradient is given according to the chronology of each group. See S6 Table for details regarding the populations considered.

The archaeological site of Gougenheim, localized in Alsace (northeast of France, 30 kilometers away from Strasbourg, Fig 2), holds a key position in the debate surrounding both the origin of the Michelsberg groups and the significance of the mortuary treatments observed. In 2009, excavations conducted in Gougenheim permitted the discovery of 30 circular pits containing 46 individuals, providing the most important human remains sample recovered from circular pits to date. Radiocarbon dating conducted on human bones ranged between 4,100 and 3,500 cal. BC and permitted confirmation of the association of the large majority of the structures to the Late Neolithic and more precisely to the Michelsberg and Munzingen cultures [33]. Located at a central point between the Paris Basin and Germany, the site thus offers a real opportunity to document the Michelsberg communities' origin.

The archaeothanatological and biological analysis of the human remains enabled the demonstration of the combined presence of mature and immature individuals, as well as a balanced number of male and female subjects [33]. Among the 46 individuals recovered in Gougenheim, 13 were found in the *CV* position and 27 in the *NCV* position, whereas the position of the seven remaining individuals could not be described. No biological difference could be detected between the subjects found in the *CV* or *NCV* position, and no specific spatial organization of *CV* vs. *NCV* structures could be observed at the site level (see Fig 1A).

All evidence gathered in Gougenheim supported more probably the sacrifice hypothesis for the interpretation of mortuary practices. Although the ritual killing of individuals can exemplify funerary accompaniment as well as sacrifice *stricto sensu*, the accompaniment hypothesis seems rather implausible in Gougenheim for several reasons. First and primarily, even if bodies lying in the *NCV* position, in isolation or in multiple burials, have sometimes been defined as “peripheral accompaniment” (i.e., individuals buried separated from the principal dead [34]), the peculiar spatial organization described in ethnological examples for such peripheral accompaniment [35] could not be detected in the Gougenheim site (see Fig 1A). Another important issue is that data compiled at Gougenheim and at the regional level suggested two separate systems of selection: an overwhelming majority of juveniles for individuals buried with the main deceased and three-quarters of adults (mostly women) buried in isolation [24]. Finally, several individuals had suffered a peri-mortem act of violence: parts of the body have been taken out of the pit or placed in a non-anatomical position, and in the case of individual 1076–1, the left femur and right tibia showed burn traces. Such practices are never recounted in cases of funerary accompaniment but have been described in ritual contexts [24, 36]. In sum, sacrifice practice as recorded in ethnographical works fits the Gougenheim archaeological dataset in many ways, including the final treatment of the victims’ bodies as ritual wastes.

To pursue the interdisciplinary investigations developed on the exceptional site of Gougenheim, we conducted a genetic analysis of 22 subjects representative of different mortuary practices (more information is provided in S1 Table). The analyses focused on both the maternal (mitochondrial DNA—mtDNA) and paternal (Y chromosome) lineages of the community. For the first time, a multi-scale study conducted at the regional and local levels permitted addressing both the question of the Michelsberg communities’ origin and dynamics, and the interpretation of their particular mortuary practices.

## Material and methods

### aDNA preparation and extraction

46 individuals were excavated from Gougenheim necropolis and are currently stored at the Center of Conservation and Archaeology at Sélestat (Alsace) and available for study. We chose 22 individuals with exploitable teeth for DNA analyses, spatially distributed in all Gougenheim archaeological site sectors and representing all types of inhumation structures (structure grouping individuals in different positions, structures grouping several CV individuals, or structure with only one NCV individual—all details about samples are present in S1 Table). While the aDNA analyses were not anticipated before the excavation, the individuals were excavated without specific aDNA care. Consequently, the teeth sampled were systematically decontaminated, i.e., scraped, cleaned with bleach and subsequently exposed to UV radiation for 20 minutes on each side. All established aDNA guidelines were then followed to minimize contamination during all subsequent steps of analyses conducted in the aDNA facilities of the UMR PACEA (Bordeaux University). To trace the source of potential human contamination, all of the persons (N = 11) in contact with the human remains (from the excavation to the aDNA lab, each researcher considered has handled all human remains) were genotyped (S7 Table). The samples were then reduced to powder and incubated overnight in lysis buffer (0.5 M EDTA, pH 8, 25mg/mL proteinase K, and 0.5% N-Lauryl sarkosyl). The procedure of Mendisco et al. [37], which uses the DNA “NucleoSpin Extract II” kit (Macherey-Nagel, Düren, Germany), was followed to extract the DNA.

### aDNA analyses

Eighteen mitochondrial SNPs and 10 Y chromosome SNPs were typed through one multiplex using MALDI-TOF MS-based SNP genotyping (iPLEX® Gold technology, Sequenom, Inc., San Diego, CA). This genotyping was conducted as previously described [37] to assess the DNA conservation for every sample and determine the mitochondrial (maternal) and Y chromosome (paternal) haplogroups. All primers used for these experiments and procedure details are available in Rivollat et al. [15]. Four overlapping fragments of the mtDNA HVR-I control region were also amplified and analyzed following the procedures described in Rivollat et al. [15] to determine the maternal haplotypes of the individuals (nps 16,024–16,380). All reported mutations were established according to the revised Cambridge Reference Sequence (rCRS) [38]. Each individual's haplogroup and haplotype were determined based on PhyloTree.org (mtDNA tree build 16 [39]). The sequences were deposited in the GenBank database (http://www.ncbi.nlm.nih.gov/genbank/; accession numbers KY485916-KY485933).

### Descriptive analyses

Sequences from all published aDNA data dating from the Mesolithic to the Late Neolithic periods were compiled (14,500–2,600 cal. BC). We selected farmers’ groups attributed to well-identified waves of Neolithization, i.e., starting with the Stärcevo groups for Central Europe and the Danubian wave of Neolithization and starting from the Cardial groups for the southern Mediterranean wave (see S6 Table). The Gougenheim group was gathered with the data published for the Michelsberg site of Bruchsal-Aue, in western Germany [23]. The global sequence dataset was divided into 16 chronological, geographical and cultural groups in order to discuss (i) the implication of different anterior groups in the Michelsberg gene pool constitution and (ii) the relationships of the Michelsberg group with contemporaneous and later populations.

**Fig 3:**
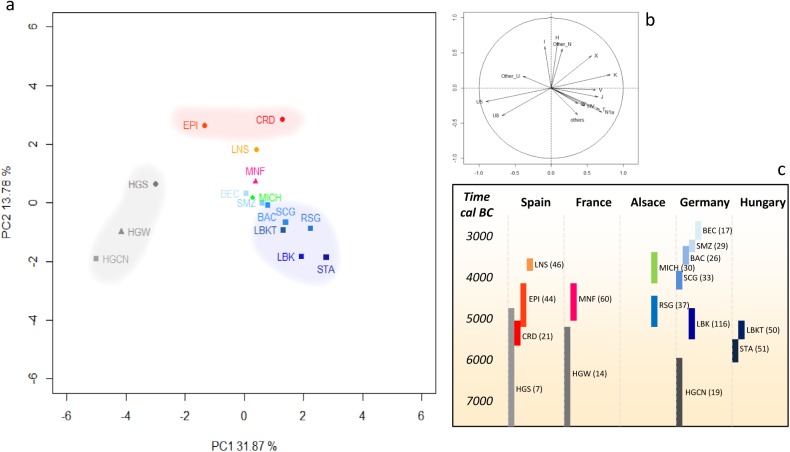
Principal component analysis (PCA) of the ancient mtDNA dataset. (a) PCA performed with haplogroup frequencies. (b) Circle of correlation. (c) Chronological overview of groups with the number of individuals between parentheses (see S6 Table for details). Colors are the same as in Fig 2. HGCN: Hunter-gatherers from Central Europe; HGW: Hunter-gatherers from West Europe; HGS: Hunter-gatherers from South Europe; STA: Starcevo-Körös; LBKT: Linearbandkeramik in Transdanubia; LBK: Linearbandkeramik; CRD: Cardial; EPI: EpiCardial Early Neolithic in Spain; RSG: Grossgartach/Planig-Friedberg/Rossen; MNF: Middle Neolithic in France; SCG: Schöningen; MICH: Michelsberg; BAC: Baalberge; LNS: Late Neolithic in Spain; SMZ: Salzmünde; BEC: Bernburg.

Haplotype diversities (S4 Table) and Fst values (S2 Table) between populations were calculated using Arlequin software (version 3.5.1.2 [40]). All HVR-I mitochondrial sequences available for nps 16,024–16,380 were used (shorter sequences were not included; N = 531). Classical PCA based on haplogroups frequencies was performed considering major European and near eastern haplogroups, that is, U5, U8, U(xU5,U8), H, HV, V, J, T, K, N1a, N(xN1a), X, I, W and others. Based on these haplogroups' frequencies, PCA was plotted using R version 3.1.2 (Pumpkin Helmet) (Fig 3) and Ward Clustering (using Euclidean Similarity Index and 5000 bootstraps) was performed with Past version 3.14 software [41] (S1 Fig). MDS based on Fst values (S2 Table) was plotted using R version 3.1.2 (Pumpkin Helmet) (S2 Fig).

We used Ancestral Shared Haplotype Analysis ASHA [42], as modified by Szécsényi-Nagy et al. [43], accounting for the temporal succession of cultures, to ascribe mtDNA haplotypes to particular cultures or time periods and to identify the number of ancestral lineages in each culture (S3 Fig). Thirteen Neolithic farmer datasets were set in a specific chronological order: STA, LBKT, LBK, RSC, SCG, MICH, BAC, SMZ, BEC for Central Europe (Hungary, Germany and Alsace) / CAR, EPI, MNF, LNS for western Europe (Spain and western France, see S5 Table). Because ancient European hunter-gatherers were represented by a very poor and certainly non-representative number of haplotypes (compared with farmers), we first considered the U/U2/U4/U5/U8 haplotypes to be "hunter-gatherer haplotypes" ("H-G" haplotypes S5 Table) [15, 44]. We then listed all haplotypes shared between ancient Neolithic groups (before 4,000 cal. BC) from both Neolithization waves (groups STA, LBKT, LBK, CAR, EPI) and considered them to be ubiquitous farmer haplotypes that were phylogeographically uninformative ("F" haplotypes, S5 Table). Each remaining lineage within a given cultural dataset was traced back to its earliest match in the chronology and regarded as an ancestral lineage that arose in this culture for the first time (S5 Table). This approach enabled us to estimate the number of mtDNA lineages directly transmitted from ancestral farmer groups to more-recent ones, thereby providing primary insight into each group’s maternal legacy and discussing the regional maternal (dis)continuity.

## Results and discussion

From the 22 analyzed individuals, 21 exploitable mitochondrial single nucleotide polymorphisms (SNP) profiles could be obtained, permitting the unprecedented characterization of 21 maternal lineages (haplogroups) for a Michelsberg group. Among these individuals, 18 complete and authenticated HVS-I sequences (or haplotypes; see the details in S1 Table) could be characterized. An exceptionally high mtDNA typing success rate (95.5% of human remains) is noteworthy for the Gougenheim site, consistent with the previous good results from the region [17]. Nevertheless, no Y chromosome SNP profile could be determined, questioning the conservation of nuclear DNA in the region. Consequently, the multi-scale analyses conducted stood on the maternal gene pool characterized for the Gougenheim group.

### Michelsberg group origin

A striking diversity of haplogroups characterized the group of Gougenheim, including H, J, K, N1a, T, W, X and U5 lineages. The maternal data gathered for the Gougenheim group (N = 21) were then grouped with the mtDNA data previously published for 9 individuals from the Michelsberg site of Bruchsal Aue in current Germany [23] and permitted the more precise description of the maternal gene pool of Michelsberg communities ("MICH" group, S6 Table). We then compared the MICH maternal pool, both at the haplogroup frequencies (PCA (S3 Table and Fig 3) and Ward Clustering (S1 Fig)) and at the haplotype composition levels (F_*ST*_ values and MDS) with ancient H-G and Neolithic groups (see S2 Table and S2 Fig for details). An ancestral shared haplotype analysis (ASHA [42]), as modified by Szécsényi-Nagy et al. [43], was also conducted to identify the number of lineages inherited from previous groups in each culture (S5 Table and S3 Fig). Both PCA and MDS evidence showed that the diversity of the MICH group fitted well with the known ancient European Neolithic population diversity (Fig 3 and S2 Fig). Nevertheless, when going deeper in the ancient groups' affinities, an important genetic discontinuity could be highlighted in Alsace / western Germany between Middle Neolithic groups culturally affiliated with Roessen and Grossgartarch ("RSG") and the Late Neolithic MICH group. This maternal rupture could be linked to important differences in terms of genetic legacy from (i) Central European farmers, (ii) western European farmers and (iii) ancient European H-G. Indeed, descriptive analyses (Fig 3 and S2 Fig) consistently localized the RSG group among the Central Europe variability (blue), along with Starčevo groups ("STA") in Hungary or *Linearbandkeramik* groups ("LBKT" in Transdanubia and "LBK" in Germany) in Central Europe [5, 17, 45, 46]. Moreover, the ASHA permitted determining that 28.1% of the RSG mtDNA haplotypes were directly derived from the STA, LBKT and LBK groups (S3 Fig). At the opposite, the MICH group stood at a clearly more distant position in relation to Central European farmers (especially obvious in the MDS, S2 Fig) and presented far fewer haplotypes (7.4%) directly derived from STA, LBKT or LBK groups. The maternal differentiation characterized between RSG and MICH can also be explained by higher maternal affinities between the MICH group and farmers from the Paris Basin (France, MNF group). Indeed, all descriptive analyses showed small genetic distances between the MICH and Middle Neolithic groups from France (Paris Basin, "MNF" group). In the Ward Clustering conducted (S1 Fig), the MICH group formed a cluster with the MNF group, and this specific cluster was supported by the most important bootstrap value. Surely correlated to these affinities with farmers from the Paris Basin, a strong H-G legacy was noted in the MICH group, slightly visible on the PCA (Fig 3) and illustrated by the high frequency of typical H-G maternal lineages (14.8%, S3 Fig). This H-G legacy is far more important than H-G legacies measured in Early and Middle Neolithic farmer groups from Hungary (STA = 4.1%, LBKT = 5.1%), Germany (LBK = 2.7%, Schöningen group "SCG" = 9.1%) and Alsace (RSG = 6.3%), but similar to what could be measured for the same periods in southwestern and western Europe (EPI = 22.7%, MNF = 14.6%). In Germany, the resurgence of a significant number of H-G lineages was observed thereafter, around 3,100 cal. BC in the context of the Bernburg culture ("BEC") (S3 Fig) [5, 11].

Taken together, our data highlighted a major genetic break between Middle Neolithic LBK-derived groups in Alsace and the subsequent Late Neolithic Michelsberg groups. This genetic discontinuity appeared to be linked to new affinities with Middle Neolithic farmers from the Paris Basin, correlated to a new and important H-G legacy. All of the evidence gathered is in favor of a close genetic connection of the Michelsberg group with farmer communities from the Paris Basin region. When chronologically and geographically replaced, the data gathered suggest that the diffusion of the Michelsberg culture was linked to a movement of human groups from West (Paris Basin) to East (Rhine region). Since an admixture between H-G and farmers maternal components has been demonstrated in the Middle Neolithic groups of the Paris Basin (Gurgy "les Noisats", 5,000 to 4,000 cal. BC [15]), we can propose that the important frequencies of H-G haplogroups measured in the MICH group may have been indirectly inherited from the MNF group in the Paris Basin (see below). The occidental origin (in the Paris Basin) of the Michelsberg culture has been proposed for a long time, based on archaeological data [20–22]. This hypothesis considered that the Michelsberg culture did not appear in the Rhine region as alternatively supposed [47, 48] but emerged in the Paris Basin from the interactions between the *Chasséen* culture, itself originating from southern France, and the Noyen culture, itself deriving from the interaction between *Cerny* (Paris Basin) and *Chasséen* (S4 Fig). The major changes observed in pottery traditions between the Michelsberg culture and anterior cultures in Alsace and Germany, combined with the diffusion of specific ceramic features from southwestern traditions in the Paris Basin, led the author to consider that migrations may have been involved in such important transformations [20]. Consistently, the paleogenetic data provided by our study supported the involvement of farmer group migration to explain such a global cultural rupture. The groups involved in the Michelsberg diffusion originated in the Paris Basin and carried in Alsace few LBK-derived maternal lineages but a significant number of Paris Basin farmers and H-G maternal legacy.

### New elements for discussing the dynamics of H-G/farmer interactions during the Neolithic period

The new mtDNA data gathered for the Michelsberg group also permit filling an important chronological and geographical gap to complete the scenario of H-G—farmer interactions during the Neolithic periods. ASHA analysis showed that important H-G legacy was first apparent in northern Spain during the Epicardial period (5,200–4,000 cal. BC, EPI = 22.7%; S3 Fig). The absence of H-G lineages in the Cardial group "CRD" may be due to the very poor quantity of data available (N = 11). Subsequently or simultaneously, such a H-G legacy appeared in the Paris Basin during the Middle Neolithic (5,000–4,000 cal. BC, MNF = 14.6%). In the meantime, very rare occurrences of H-G lineages could be observed in Central Europe, in accordance with recent genomic data [11]. The available mtDNA data then support the scenario of an increasing H-G admixture into farmers’ groups migrating farther West in Europe (in Spain and France) during the Neolithization processes. This hypothesis has already been proposed through the analysis of the maternal gene pool of the Paris Basin Middle Neolithic farmers [15] and has been recently reinforced by coalescent simulations [49].

At the beginning of the Late Neolithic, the important H-G legacy was still visible in Spain (3,700–3,500 cal. BC, "LNS" = 25.6%), whereas the MICH group was the only group in Central Europe to present a significant H-G maternal lineage frequency (14.8%). The first noticeable resurgence of H-G maternal lineages in Central Europe occurred only at the end of the fourth Millennium, in the context of the Bernburg culture (BEC = 29.4% [5, 11, 44]). This H-G legacy resurgence in Central Europe has already been largely described [5] and is illustrated in our PCA by the progressive and chronological displacement of Central Europe farmer groups toward H-G groups. The H-G legacy resurgence observed in the Bernburg context has been linked to the important admixture demonstrated between H-G and farmers in Scandinavia (in the North European Plain), one millennium earlier, in the context of the emergence of the Funnel Beaker Culture (TRB or TBK) [3, 4, 50]. The Bernburg groups, a late representative of the TRB groups in Central Europe, must have inherited their important frequencies of H-G haplogroups from their northern ancestors. Nevertheless, a recent genome-wide study conducted on 69 ancient Europeans [11] has provided arguments in favor of a different source of the H-G legacy resurgence detected in Central Europe. Indeed, the H-G component found for individuals from Central Europe and dated from the fourth Millennium showed more genomic affinities with H-G from western Europe (including La Braña 1- remains from Spain [51], Loschbour—H-G from Luxembourg [12] and KO1 H-G from Hungary [10]) than with H-G groups from eastern or northern Europe. Even if the genomic data available for the European H-G are still scarce and the exact source of H-G ancestry is consequently not fully established yet, these data suggest two possible sources of H-G legacy resurgence in Central Europe, *i*.*e*., from western Europe H-G and northern Europe/Scandinavian H-G. Evidently, further genomic analyses on H-G and Early/middle Neolithic farmers are necessary to delineate the process of H-G—farmer interactions during the Neolithic period in Europe, as well as in the regions discussed in the present paper. However, the gap observed between the appearance of the H-G legacy in Spain and in France between 5,000 and 4,000 cal. BC and the noticeable resurgence of the western H-G legacy in Central Europe from the fourth Millennium BC [5, 44] could be partly filled, both chronologically and geographically, by our Michelsberg group. Indeed, the migration of Michelsberg groups originating from the Paris Basin and carrying an important H-G legacy in Alsace and Germany, might have contributed to the subsequent resurgence of western H-G ancestry in these regions. Since previous analyses have demonstrated that the MNF group had inherited a noticeable fraction of its maternal gene pool from descendants of farmers associated to the Mediterranean neolithisation wave [47], we cannot exclude that part of the H-G legacy measured in MICH group finds its origin in southwestern farmers groups.

### Gougenheim's mortuary practices

Among the 21 individuals providing mitochondrial profiles, 9 were found in the *CV* position, whereas 12 were found in the *NCV* position (Fig 1B). Only 2 genotyped individuals originated from some possible multiple “asymmetrical” deposit (structure 1029 grouping individual 1029–1 in the *CV* position and 1029–2 in the *NCV* position, but at two distinct levels), when all remaining genotyped individuals found in the *NCV* position originated from multiple “non-conventional” deposits and all remaining genotyped individuals found in the *CV* position originated from individual “conventional” deposits. Both the *CV* and *NCV* groups showed lineages originating from the (south)western regions as described above. Whereas all "H-G lineages" (of potential western origin) were found concentrated in the *NCV* group, the *CV* group contained a strong proportion of haplogroups H (H, H1 and H3) and X, which were more common in southern European and Paris Basin farmers (Fig 3). A similar phylogeographic signature could then be observed in both the *CV* and *NCV* groups, indicating a unique cultural and biological group at the local level but differently treated in death. Interestingly, isotope analyses demonstrated diet homogeneity between both groups and, as such, supported a shared geographical origin [52].

If we highlighted that the Gougenheim group represented a phylogeographically consistent biological group, we note differences in maternal gene pools of sub-groups *CV* and *NCV*. Although the small number of individuals in each group (and the very important mitochondrial diversity measured in each group, see below) did not allow any satisfactory statistical testing of differentiation, we could see that haplogroups W and X were specific to group *CV*, whereas haplogroups N1a, U5 and T were specific to group *NCV* (Fig 1B). Only two HVR-I haplotypes were found to be shared by both the *CV* and *NCV* groups: haplotypes J1_16069–16126 and H_CRS. Nevertheless, these haplotypes are very frequent in Neolithic populations (found respectively at 6.7% and 14.3% in the compiled database) and thus represent a poor indicator of group affinities or maternal kinship. We consider that the distinct maternal gene pools characterized for both the *CV* and *NCV* groups, submitted to distinct mortuary treatment, points to a relative genetic isolation of the two groups from each other. This genetic isolation may have been related to the social stratification of the community, with combined endogamy in each social group and the genetic isolation of distinct social stratum and/or to some geographic distance between separate communities. These assumptions nicely echo the hypothesis of sacrifices retained for the Gougenheim site. Ethnological and archaeological examples indeed show that the victims of human sacrifice are often slaves, sometimes specifically bought for the occasion, but more regularly abducted during ambushes or razzia in some enemy villages of the neighborhood or taken into captivity during warlike events [24]. In such circumstances, a genetic differentiation between distinct social groups corresponding to master *vs*. slave groups or between adversarial neighbor groups (allied communities are characterized by the exchange of women and genes) can be expected. In consequence, the obtained paleogenetic data fit well with the sacrifice hypothesis, even if they cannot be conclusive. However, the “peripheral accompaniment” assumption—archaeologically unlikely in Gougenheim—involving the same social stratum of subjugated people, is theoretically possible when considering only DNA data.

Both sub-groups *CV* and *NCV* presented an important maternal diversity (0.83 +/- 0.13 and 0.92 +/- 0.09, respectively), in total accordance with mitochondrial diversities generally measured for the Neolithic period in Europe [15, 17, 53] (S4 Table). As already proposed for other Neolithic farmer groups [43, 53, 54], this maternal diversity could be an indicator of a patrilocal social system (although Y chromosome data are necessary to directly test this hypothesis). Interestingly, each multiple structure also showed a striking maternal diversity. For instance, the structures 1076 and 1077, grouping 4 *NCV* individuals each, presented a total of 6 distinct mitochondrial sequences over the 7 individuals genotyped (S1 Table). Moreover, when considering all of the structures with aDNA data, no grouping or spatial structure could be highlighted according to maternal lineages (Fig 1A). Finally, concerning the individuals found in multiple “non-conventional” deposits, no shared haplotype could be detected. Only two individuals found in the multiple “non-conventional” structure 1077 shared the haplotype K_16224–16311, but once again, this very common haplotype in Neolithic groups (2.02%) may not be indicative of a maternal kinship. Considered together, the mtDNA data compiled for the Gougenheim site project the image of the random sampling of individuals inside both sub-groups *CV* and *NCV*. The biologically and socially differentiated sub-groups, submitted to very different mortuary treatments, must have been large enough to present such maternal diversities. Even if the presented arguments cannot be conclusive, the picture depicted at the genetic level is in total accordance with the idea of the random kidnapping of the individuals submitted to sacrifice. Whether these individuals were neighbors randomly abducted for religious needs or were slaves remains an open question.

## Conclusions

Our study demonstrates the need for multi-scale aDNA analyses to disentangle the complex relationships between the cultural and biological evolution of human populations. The genetic analyses conducted on the exceptional site of Gougenheim, grouping the most important sample of human remains associated with the Michelsberg culture and with mortuary practices involving inhumations within circular pits, have shed light on the group origin and social organization. First, at the regional/continental scale, the mitochondrial data recovered support the Paris Basin as the origin of the groups affiliated with the Michelsberg culture. We then propose that the cultural diffusion of ceramics associated with the Michelsberg has been directly linked to the migration of human groups. These groups, originating in the Paris Basin and established in Alsace and Germany during the Late Neolithic, carried not only a significant West European farmer legacy but also an ancient European hunter-gatherer legacy. This migration may thus have represented a landmark in the progression of the hunter-gatherer legacy from the Paris Basin to Alsace / Western Germany at the beginning of the Late Neolithic. The clear entanglement of biological and cultural diversities has also been demonstrated at the local level. Indeed, we could demonstrate that the individuals deposited in the non-conventional position in circular pits presented distinct maternal lineages than the individuals found in conventional position and may represent a special group within the community. The relative genetic isolation of this specific group nicely echoes both its social distinction and the hypothesis of sacrifices retained for the Gougenheim site. Undoubtedly, the obtaining of genomic data for the Gougenheim group should provide conclusive arguments concerning the origin of the Michelsberg groups and the understanding of their mortuary practices. To date, genomic data have been published for relatively isolated individuals, chronologically and geographically dispersed (and maybe not fully representative of ancient culture and group diversities), to provide an unprecedented large-scale and long-term evolutionary scenario. Keeping in mind the need for multi-scale approaches to disentangle biological and cultural processes, we hope that the community level will soon benefit from this powerful paleogenomic approach.

## Supporting information

S1 Fig Ward clustering. Dendrogram obtained with Past version 3.14 software, using Euclidean Similarity Index and 5000 bootstraps.
(PDF)

S2 Fig Multidimensional Scaling (MDS). MDS performed on the F_st_ values (S2 Table).
(PDF)

S3 Fig Ancestral Shared Haplotype Analysis. ASHA performed on the Neolithic HVR-I sequences, combined with frequencies of Hunter-Gatherers haplogroups (See S5 Table for the analysis details). Grey squares indicate groups presenting more than 10% of Hunter-Gatherers legacy.
(PDF)

S4 Fig Chrono-cultural overview from Paris Basin to Rhine region from 4600 to 3850 cal BC. (PDF)

S1 Table Individual data for the 22 analyzed samples. (XLSX)

S2 Table Fst values for ancient dataset. (XLSX)

S3 Table Haplogroup absolute frequencies. (XLSX)

S4 Table Haplotype diversities. (XLSX)

S5 Table Results of ASHA conducted on the Neolithic haplotypes, combined with frequencies of hunter-gatherers haplogroups. (XLSX)

S6 Table List with all references discussed in the text giving relevant information in chronological order. (XLSX)

S7 Table Summary of the HVR1 sequences for the excavation and genetic teams. (XLS)
