# Supplementary figures and images for: Multi-scale ancient DNA analyses confirm the western origin of Michelsberg farmers and document probable practices of human sacrifice
Source: PLoS One. 2017 Jul 5;12(7):e0179742. doi: 10.1371/journal.pone.0179742 (PMC5497962; doi:10.1371/journal.pone.0179742)

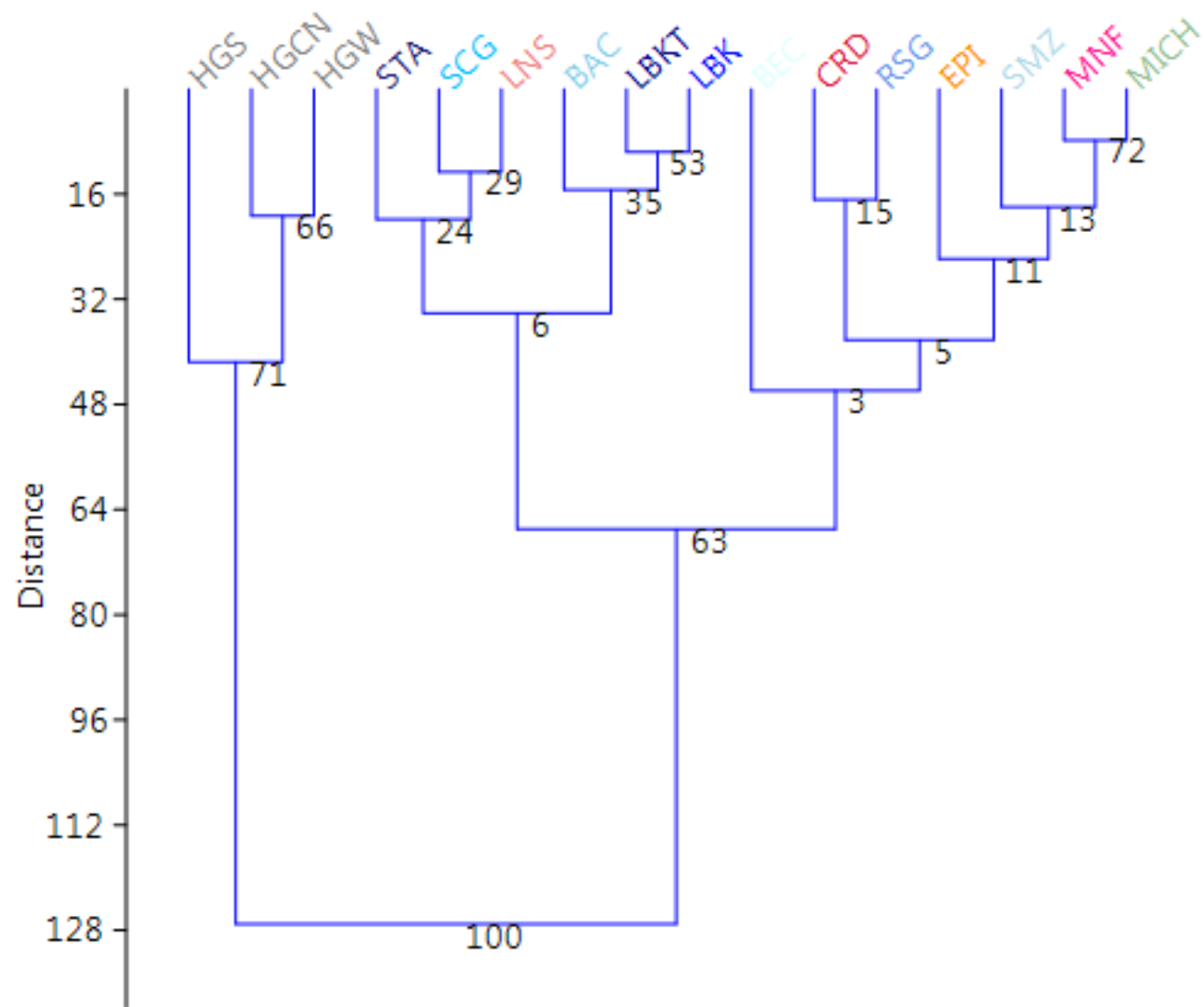

Supplement: S1 Fig — Dendrogram obtained with Past version 3.14 software, using Euclidean Similarity Index and 5000 bootstraps. (PDF) [file pone.0179742.s001.pdf]

# MDS performed on pairwise FST

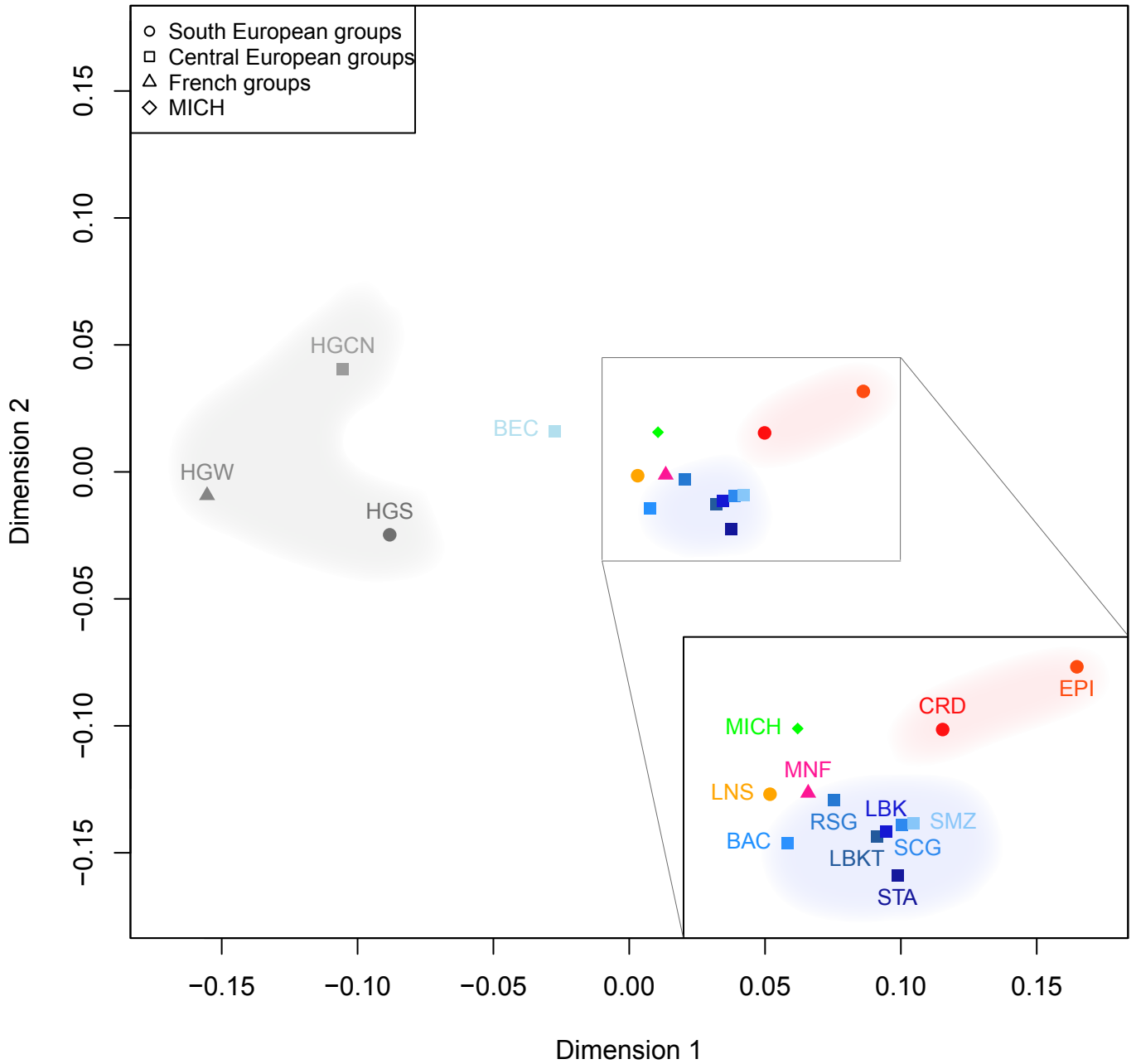

Supplement: S2 Fig — MDS performed on the Fst values (S2 Table). (PDF) [file pone.0179742.s002.pdf]

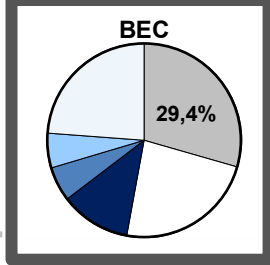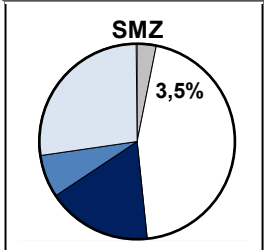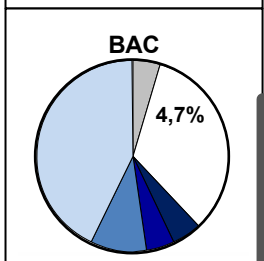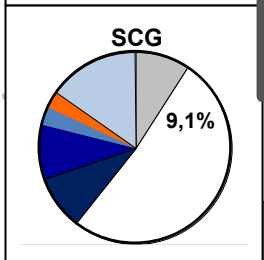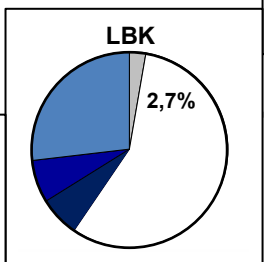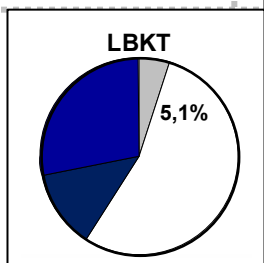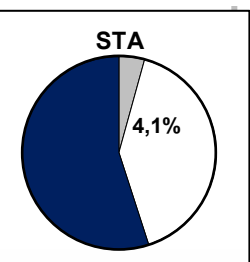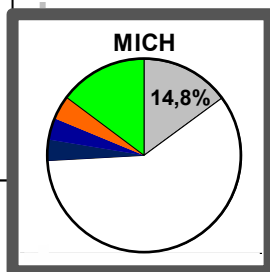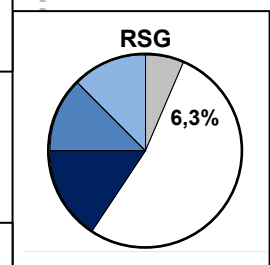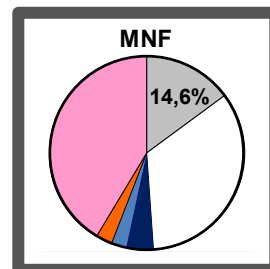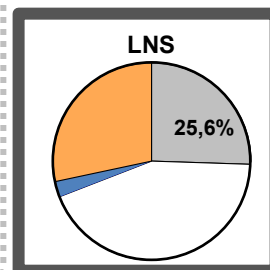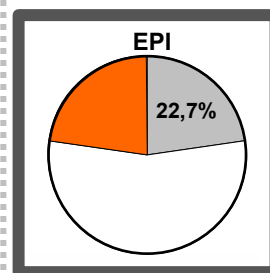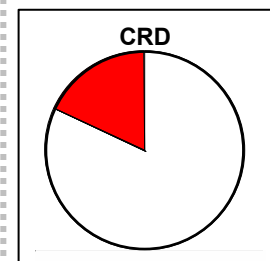

3000 BC

4000 BC

5000 BC

6000 BC

HUNGARY

GERMANY

ALSACE

PARIS  
BASIN

SPAIN

Supplement: S3 Fig — ASHA performed on the Neolithic HVR-I sequences, combined with frequencies of Hunter-Gatherers haplogroups (See S5 Table for the analysis details). Grey squares indicate groups presenting more than 10% of Hunter-Gatherers legacy. (PDF) [file pone.0179742.s003.pdf]

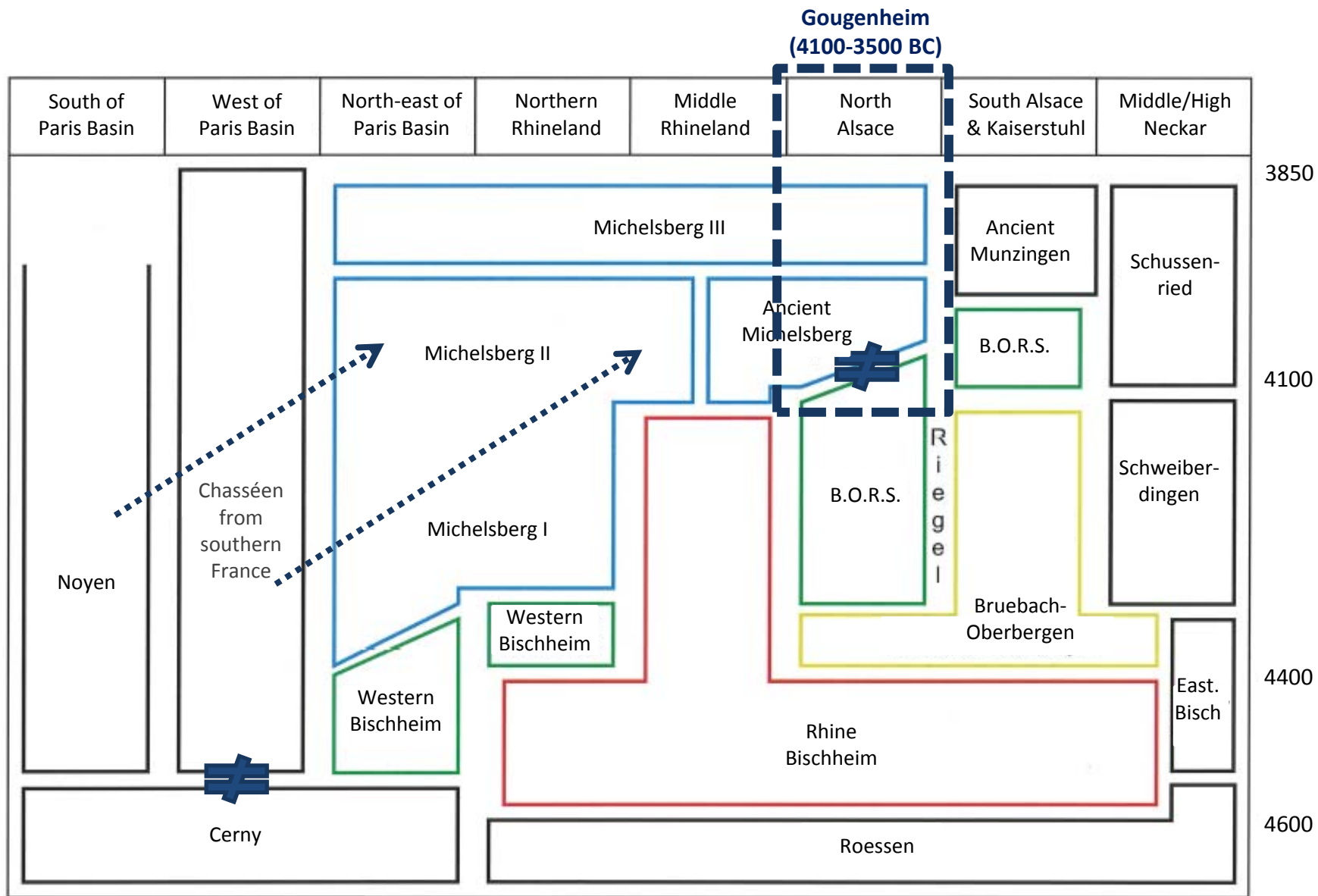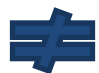

Cultural discontinuity

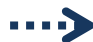

Cultural influences

After Jeunesse 2002, adapted with Jeunesse 1998

Supplement: S4 Fig — (PDF) [file pone.0179742.s004.pdf]
